# The unique activity of the bone morphogenetic protein TGH4 affects the embryonic development of *Trichinella spiralis* and the establishment of vaccine protection

**DOI:** 10.1186/s13567-025-01473-4

**Published:** 2025-02-07

**Authors:** Wenjie Shi, Yi Liu, Yan Liu, Xue Bai, Yue Liang, Yaming Yang, Fangwei Wu, Mingyuan Liu, Ning Xu

**Affiliations:** 1 State Key Laboratory for Diagnosis and Treatment of Severe Zoonotic Infectious Diseases, Key Laboratory for Zoonosis Research of the Ministry of Education, Institute of Zoonosis, and College of Veterinary Medicine, Jilin University, Changchun, 130062 China; 2 College of Food Science and Engineering, Jilin University, Changchun, 130062 China; 3 College of Public Health, Jilin Medical University, Jilin, 132013 China; 4 Department of Helminth, Yunnan Institute of Parasitic Diseases, Puer, China

**Keywords:** *Trichinella spiralis*, bone morphogenetic protein, vaccine, immunoregulation, protective efficacy

## Abstract

In recent years, animal outbreaks of trichinellosis have been reported in Mexico, China, Algeria, Croatia and others, which is a challenge to meat safety and public health. Vaccination is an important means to control trichinellosis, but one of the main challenges in vaccine development is screening for protective candidate antigens. Bone morphogenetic proteins (BMP)), which are potential vaccine research targets, play key roles in the growth and development of metazoans. The BMP homologue TGH4 was identified from *Trichinella spiralis* (*T. spiralis*), and recombinant C-terminal mature rTGH4-m had BMP activity and exerted regulatory effects on both mouse fibroblast and *T. spiralis* embryonic development. The construction of a protein mutant without activation potential confirmed that BMP activity had a negative regulatory effect on the establishment of immune protection by affecting the innate, adaptive, and humoral immunity of mice. Stripping protein activity can enhance immune protection and host resistance to *T. spiralis*. Our findings demonstrate that the TGH4 mutant is an important vaccine candidate antigen that blocks embryonic development, kills larvae, and provides insight into parasite vaccine research.

**Supplementary Information:**

The online version contains supplementary material available at 10.1186/s13567-025-01473-4.

## Introduction

Trichinellosis is a zoonotic parasitic disease of the digestive tract that can ultimately establish long-term parasitism in the muscle tissue of multiple animals [1]. Several outbreaks of trichinellosis in humans and animals have been reported in recent years, including in southwestern China [2], Mexico [3], Croatia [4], Romania [5], Arizona, Minnesota, and South Dakota in the United States [6]. Pork was the main cause of infection in China, whereas 32 patients in Croatia and the United States were infected through wild animals [2, 4, 6]. Currently, there are limited ways to prevent trichinellosis in domestic animals because of the strong adaptability of worms to changes in the environment. Albendazole is a highly effective nematode repellent that can reduce *Trichinella spiralis* (*T. spiralis*) adult worms in the gut but is less effective in muscular larvae (ML) with cyst protection [7, 8]. In addition, the usage of albendazole increases the possibility of drug residues in meat products, which is a potential threat to food safety. In contrast, vaccination is a safe and cost-effective method to quickly help animals develop long-term resistance to pathogenic infections [9, 10]. Both gamma-irradiated ML and the freeze-thawed whole NBL showed good immune protection efficacy, and the reduction rate of the ML load was greater than 70%, but they presented high production costs and a high risk of *T. spiralis* parasitism [11, 12]. Hence, the development of low-cost protein vaccines has become important. One of the greatest challenges in establishing great immune protection with protein vaccines is screening suitable protein molecules.

Nematodes can successfully complete their life cycle in different hosts by a sophisticated parasitic strategy [13, 14], which provides a reference for vaccine research. Currently, the screening of candidate antigens for nematode vaccines has focused on three main directions: (I) key effector proteins related to growth and development, (II) key effector proteins related to the regulation of the host immune system, and (III) key effector proteins related to metabolism [15]. Bone morphogenetic protein (BMP) family proteins play important roles in osteogenesis, skeletal development, embryonic differentiation and maintenance of physiological homeostasis [16–18]. The active region of BMP is conserved across different species [19]. Some studies have identified BMP homologues from parasites, such as *Schistosoma japonica* and *Schistosoma mandi*, and speculated that these homologues may play important roles in the growth and development of parasites and the reproduction of offspring [20–22]. Loss of BMP signalling in *Caenorhabditis elegans* results in a smaller body size and defects in the male mating structure, which suggests that the regulation of growth and development by BMP signalling is important for nematodes [23]. In addition, the BMP homologue signalling pathway of *Echinococcus multilocularis* can be activated by human BMP2, indicating that the active sites of the BMP homologues of *Echinococcus multilocularis* and humans may be conserved [24].

This study identified the BMP homologue transforming growth factor homologue 4 (TGH4) from *T. spiralis*, which consists of 431 amino acid residues (aa) containing the signal peptide, the cleavage site of furin, and the highly conserved active mature region TGH4-m. rTGH4-m has BMP regulatory effects in mammals, and the growth and development of *T. spiralis* embryos require BMP signalling. In addition, we found that BMP activity can affect the protection efficiency of active immunity, resulting in differences in resistance against *T. spiralis* parasitism. Therefore, TGH4 is a potential vaccine candidate antigen associated with growth and development, and stripping BMP activity can increase its immune effectiveness and host resistance.

## Materials and methods

### Bioinformatics analysis of TGH4

Biological information, including the molecular weight, signal peptide, and furin cleavage site, of TGH4 (GenBank: FJ513374.1) was predicted. Spatial structural prediction of the protein was performed [25–29]. Amino acid sequence alignment of the active regions of BMPs from multiple species and evolutionary trees were generated with MEGA 11 software.

### Expression of recombinant protein

Recombinant TGH4 and TGH4-m were expressed by *E. coli* BL21(DE3) and subjected to nickel column affinity chromatography via a ÄKTA pure instrument (Cytiva). Proteins were quantified with a BCA protein assay kit, and the same molar masses of rTGH4 and rTGH4-m were used in the experiment. A mutant protein (TGH4-d) without the furin cleavage site was constructed and inserted into the expression vector Pet-28a. rTGH4-d was expressed and purified as previously described.

### Animal experiments

Six- to eight-week-old BALB/c mice and SD rats were used in the experiments and procured from Jilin University Experimental Animal Center (Jilin, China). The animal experiments were performed in accordance with the guidelines for the care and use of laboratory animals [30], and the details are as follows. All the animals were provided sufficient water and food with a 12 h light/dark cycle in a specific pathogen-free environment. To study the immune protection of proteins, five healthy mice were randomly assigned to each group and kept in a cage, including three control groups (blank control, PBS + *T. spiralis*, Adjuvant + *T. spiralis*) and two experimental groups (rTGH4/adjuvant + *T. spiralis*, rTGH4-d/adjuvant + *T. spiralis*). To collect adults at different times and newborn larvae (NBL), SD rats orally infected with *T. spiralis* (ISS534) were euthanized on days 3 and 6 of infection for intestinal collection. To investigate the effect of serum on the parasitism of migrating NBL, five healthy mice were randomly assigned to each group (four experimental groups: PBS control, negative control serum, anti-TGH4 serum, and anti-TGH4-d serum) and kept in a cage.

### Active immunity of the protein

The mice were first immunized with a 100 μL mixture of 50 μg protein and isovolumetric complete Freund’s adjuvant on day 1. A 100 μL mixture of 50 μg protein and incomplete Freund’s adjuvant was used to enhance immunity on days 14 and 28. PBS and Freund’s adjuvant were used as negative controls. The serum was collected from the tail vein of the mice at different time points. For immune protection evaluation, protein-immunized mice were infected with 250 T*. spiralis* (ISS534) on day 35. All the mice were euthanized on day 70, and the larvae in the muscle tissue were counted.

### Parasite experiment in vitro

*T. spiralis* (ISS534) obtained from infected rats in our laboratory was used in the experiments. Adult worms 3 days after infection (Ad3), 6 days after infection (Ad6), ML, and NBL were collected according to previous methods [31, 32]. Briefly, the intestinal tracts of infected SD rats were soaked in sterile normal saline supplemented with 200 U/mL penicillin and 200 mg/mL streptomycin at 37 ℃ for 3 h, and adult worms were obtained via the precipitation method. Adults were cultured with 1640 RPMI containing 200 U/mL penicillin and 200 mg/mL streptomycin at 37 ℃ and 5% CO_2_. Considering the difference in the molecular weight between the two proteins (rTGH4 and rTGH4-m) and the consistency in the number of protein molecules, the mole mass was used to calculate the amount of protein. Hence, 500 Ad3 adults were incubated with the same molar masses of rTGH4 and rTGH4-m proteins (42 ng/mL rTGH4 and 10 ng/mL rTGH4-m; 84 ng/mL rTGH4 and 20 ng/mL rTGH4-m;) or mouse serum immunized with protein three times (negative serum and anti-TGH4 serum (dilution ratio, 1:100, 1:200)) for 3 days in vitro, and the number of NBL was then detected. The culture plates of each treatment group were placed under an inverted microscope for observation. Dead NBL were straight and inactive, whereas live NBL are active [33]. After the mixture was mixed with a pipette, 20 μL of liquid was removed, and the numbers of NBL and adults were counted. Finally, the NBL was converted to the number of larvae in the total volume, and the ratio of NBL to adults was calculated.

In the NBL infection experiment, Ad6 adults were cultured to produce NBL in vitro. Five thousand Ad6 adults were incubated with PBS or mouse serum supplemented with protein three times (negative control serum, anti-TGH4 serum, and anti-TGH4-d serum (dilution ratio, 1:100)) for 16 h at 37 ℃ and 5% CO_2_ and NBL was then collected via a 100 μm mesh filter. 40 000 NBL were used to infect mice through the tail vein. The mice were euthanized after 25 days of infection and larvae in muscle tissue were counted.

### RT-qPCR

Total RNA was collected from *T. spiralis* at different time points post-infection. The PerfectStart Uni RT & qPCR Kit (TransGen Biotech) was used to reverse transcribe RNA into cDNA according to the manufacturer’s instructions. The transcription level of Tgh4 was assessed with specific primers and analysed via the ΔΔCt method. The primers for Tgh4 and *Ts*-GAPDH are listed in Table 1.

**Table 1:** **Specific primer sequences**

	Gene	Primer sequence (5’ → 3’)
RT-qPCR	Tgh4	F: CTGATGAATCCTGGTCGCGT
		R: GTGGCAACCGCAGGTCTT
	*Ts*-GAPDH	F: GTGCTGATTACGCTGTTG
		R: CTAAGCCATTGGTAGTGC

### Cell migration and proliferation of fibroblasts

The L929 mouse fibroblast line was cultured with MEM containing 10% fetal bovine serum, 100 U/mL penicillin, and 100 mg/mL streptomycin at 37 °C and 5% CO_2_. A total of 2 × 10^5^ cells were seeded into a 6-well plate and cultured for 12 h at 37 °C and 5% CO_2_. When the cells were 80 to 90% confluent, a wound healing assay was performed. Sterile PBS was used to wash the cells three times, and the sterile tip of the 200 μL yellow pipette tip was used to scratch the cells. The cells were subsequently cultured with MEM containing 100 U/mL penicillin and 100 mg/mL streptomycin. The same molar mass of protein (42 ng/mL rTGH4 or 10 ng/mL rTGH4-m) was used to incubate the cells. The number of cell scratches was recorded via microscopy at 0, 6, 12, and 24 h. The images were analysed via ImageJ software and GraphPad Prism 5 software.

A total of 1 × 10^8^ cells were stained with 5 µM carboxyfluorescein succinimidyl ester (CFSE) in 1 mL of PBS for 20 min at 37 °C in the dark. Five milliliters of MEM containing 10% fetal bovine serum was added to stop the staining. The cells were collected by centrifugation at 1500 rpm for 10 min. A total of 2 × 10^5^ stained cells were seeded into a 6-well plate and cultured with MEM containing 10% fetal bovine serum, 100 U/mL penicillin, and 100 mg/mL streptomycin. The same molar mass of proteins (42 ng/mL rTGH4 and 10 ng/mL rTGH4-m) was added to stimulate the cells for 12 h at 37 °C and 5% CO_2_. The cells were collected and assessed with a BD FACSCalibur flow cytometer.

### Western blot

Fibroblasts were separately stimulated with 42 ng/mL rTGH4 or 10 ng/mL rTGH4-m for 12 h at 37 °C and 5% CO_2_. The cells were lysed with RIPA buffer supplemented with PMSF (1 mM), and the protein in the cell lysis supernatant was quantified with a BCA protein assay kit (Solarbio Life Science). Twenty-five micrograms of protein was mixed with protein loading buffer. The protein samples were separated by SDS-PAGE or native-PAGE and transferred to PVDF membranes. The membranes were incubated with 5% skim milk for 2 h at 37 °C. Then, the diluted primary antibody was incubated with the membrane overnight at 4 °C. PBST buffer was used to wash the membrane 5 times, and the HRP-labelled anti-rabbit IgG antibody or HRP-labelled anti-mouse IgG antibody was incubated with the membrane for 2 h at 37 °C. After 5 washes with PBST, electrochemiluminescence (ECL) western blotting substrate (Beyotime) was added to detect the target protein bands. Protein expression was quantified and analysed via ImageJ and GraphPad Prism 5 software.

### Evaluation of the protection efficiency of TGH4 and TGH4-d

After the protein-immunized mice infected with *T. spiralis* were euthanized, the diaphragm and carcass without the fur or gut were collected to evaluate protection efficiency. The diaphragm was fixed with 4% paraformaldehyde at 4 ℃ for 24 h and embedded in paraffin. Sections were cut and subjected to haematoxylin‒eosin (H&E) staining. The stained sections were observed with a microscope at different magnifications. Carcass samples without fur or guts were used to collect ML, and the number and state of ML were detected.

### Detection of specific antibodies and cytokines in the serum

Specific antibodies in the serum at different dilutions were detected via indirect ELISA to analyse the antibody levels after three immunizations. A total of 0.5 μg of recombinant proteins (rTGH4 and rTGH4-d) at a volume of 100 μL was added to a 96-well plate at 4 ℃ for 12 h. The 96-well plates were blocked with 5% skim milk solution at 37 ℃ for 2 h and incubated with diluted serum at 37 ℃ for 1 h and with HRP-conjugated anti-mouse IgG, IgG1, IgG2a, and IgA (Abcam) at 37 ℃ for 1 h. For the reaction of TMB substrates, antibody levels were quantified by measuring the absorbance at 405 nm. When the OD value of the diluted serum was 2.1 times greater than the OD value of the negative control serum, it was considered positive. Cytokines, with the exception of TGF-β1, which was detected via an ELISA kit (Proteintech) according to the manufacturer’s instructions, were detected via Luminex.

### Flow cytometry

After the protein-immunized mice were infected with *T. spiralis* for 35 days, the mice were euthanized, and the peritoneal cavity cells and spleen cells were collected. The cell suspension was prepared by eliminating erythrocytes and passing it through a 70 μm mesh filter. For spleen cells, 1 × 10^6^ cells in a 100 μL volume were stained with antibodies against cell surface molecules for 30 min (PerCP/Cyanine5.5-conjugated anti-CD45 (BioLegend), APC-conjugated anti-B220 (BioLegend), FITC-conjugated anti-CD4 (BioLegend), PerCP/Cyanine5.5-conjugated anti-CD3 (BioLegend), and PE-conjugated anti-CD8 (BioLegend)). After the cells were treated with 4% paraformaldehyde for 30 min and 0.1% Triton X-100 for 40 min, they were stained with antibodies against intracellular molecules for 30 min (PE-conjugated anti-Foxp3 (BD Biosciences)). The peritoneal cavity cells were blocked with Fc receptor blocking antibodies (BD Pharmingen) for 10 min and then stained with the appropriate antibodies for 30 min (APC-conjugated anti-F4/80 (BioLegend), FITC-conjugated anti-CD16/32 (BioLegend), and PE-conjugated anti-CD206 (R&D Systems)).

### Statistical analysis

All the data were analysed and output as the mean ± standard deviation (SD) via GraphPad Prism 5 software. The data were first tested for normal distribution with normality and lognormality tests. To assess differences between various groups, one-way ANOVA, two-way ANOVA, and unpaired t tests were employed. * and # are used to show the differences in the results of different comparison methods. *, #*P* < 0.05; **, ##* P* < 0.01; ***, ###* P* < 0.001; ns: no significant difference.

## Results

### Bioinformatics analysis of TGH4 and TGH4-m

**Figure 1:**
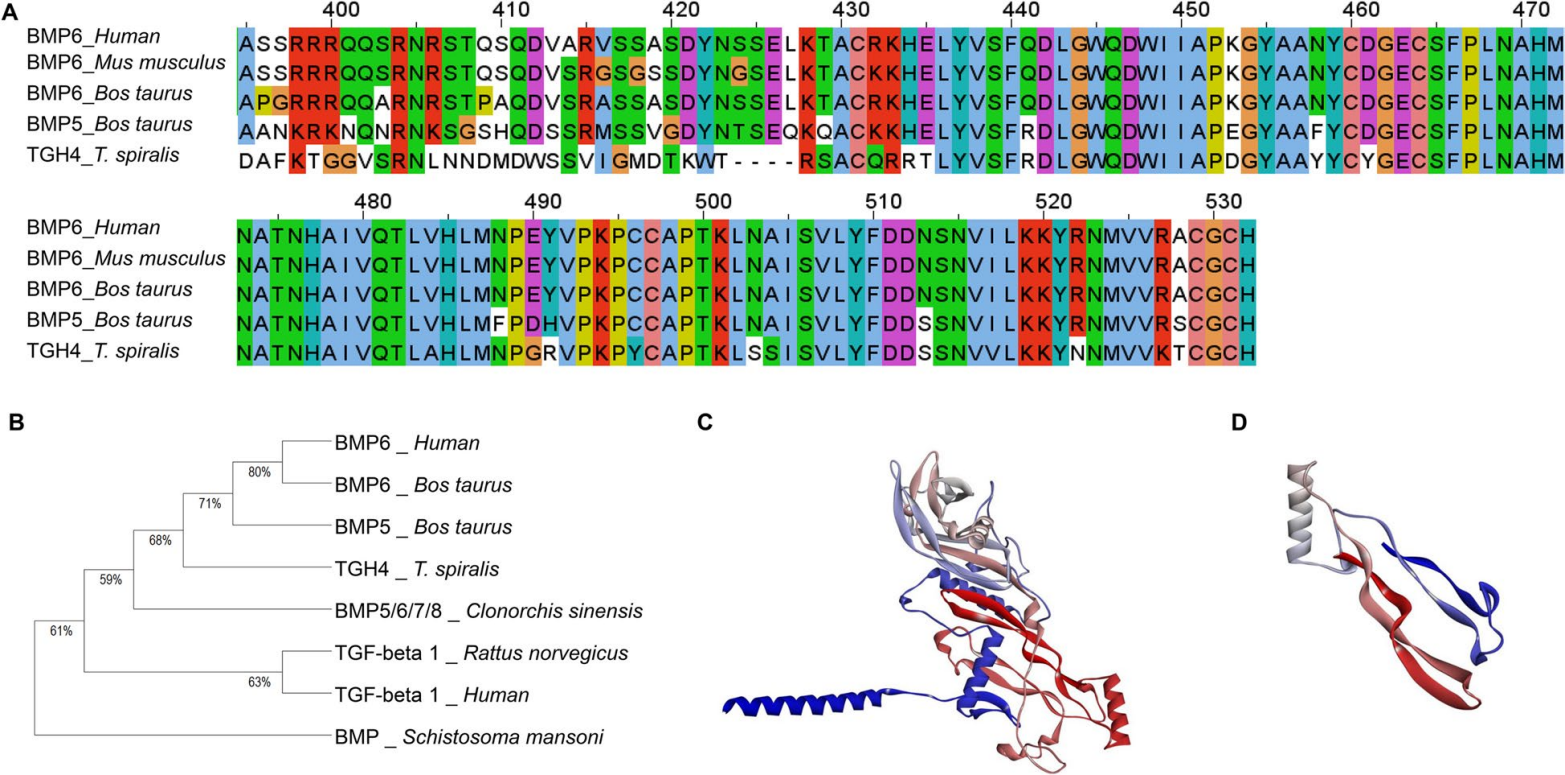
**Bioinformatics analysis of TGH4 and TGH4-m.** **A** Multiple sequence alignment of TGH4 with mammalian BMP homologues. **B** Phylogenetic relationships between TGH4 and TGF superfamily homologues. Three-dimensional spatial structure of TGH4 (**C**) and TGH4-m (**D**).

The sequence of full-length TGH4 consists of 431 amino acid residues (aa) containing the signal peptide, the cleavage site of furin, and the C-terminal active region TGH4-m (Additional file 1). In accordance with the typical characteristics of the BMP family, the amino acid sequence of the active mature TGH4-m was highly conserved (Figure 1A). In addition, the phylogenetic tree of full-length TGH4 and TGF-β superfamily proteins revealed that TGH4 clustered with mammalian BMPs, such as *human* BMP6, *Bos taurus* BMP6, and *Bos taurus* BMP5 (Figure 1B). The 3D structure of TGH4 was acquired from the UniProt server (PDB ID: D2DEC9), and the 3D structure of TGH4-m was predicted by the SWISS-MODEL server (Figures 1C and D). The best template was human BMP6 (PDB ID: 6omo), and the GMEQ was 0.84, which confirmed the high quality and confidence of the predictive model (Figure 1D).

### Expression of recombinant proteins and detection of protein activity

**Figure 2:**
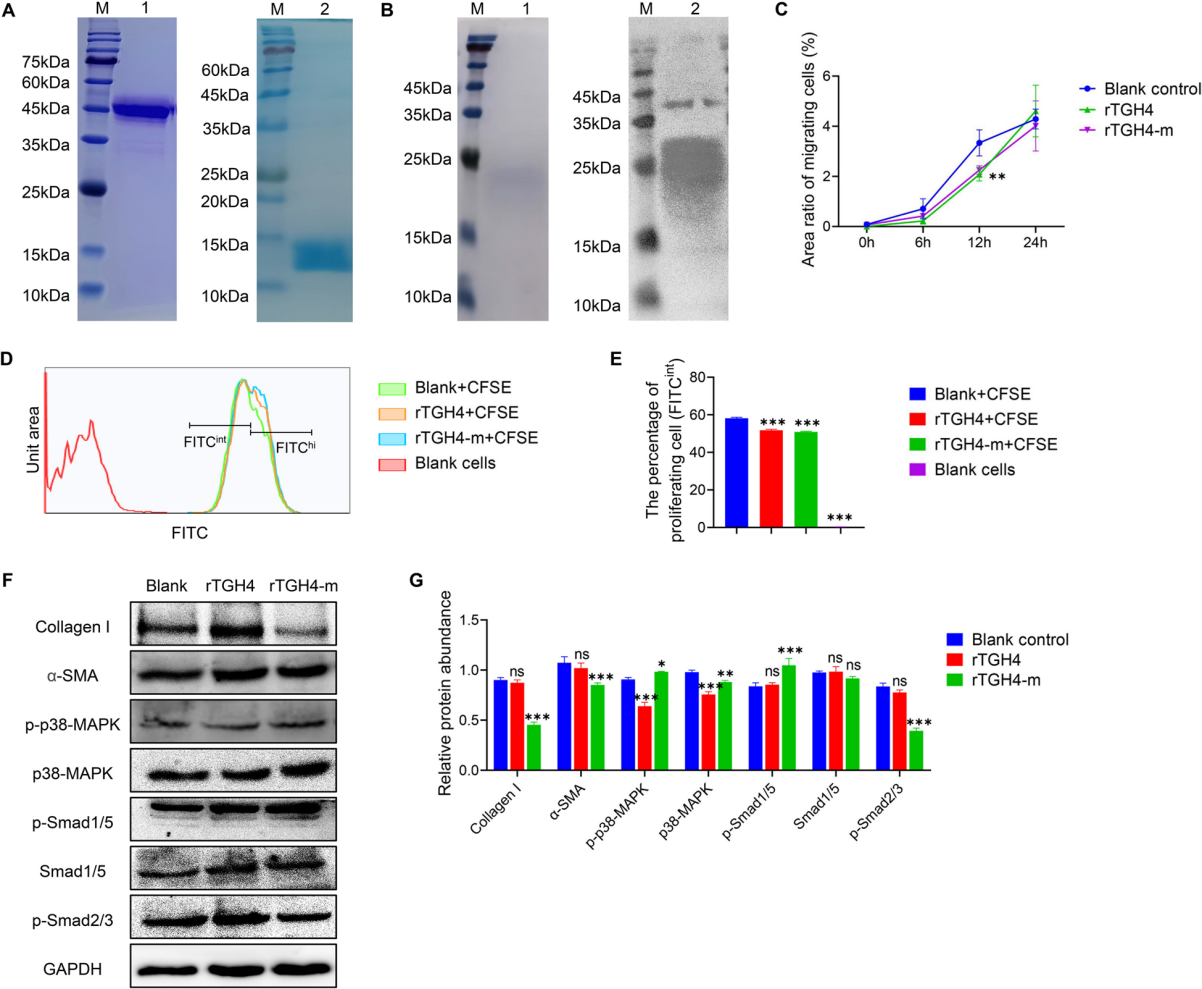
**Detection of the regulatory effects of recombinant proteins on fibroblasts.** **A** SDS-PAGE images of rTGH4 and rTGH4-m. Lane M: protein standard; Lane 1: purified rTgh4; Lane 2: purified rTGH4-m. **B** Native PAGE image and western blot images of rTGH4-m. Lane M: protein standard; Lanes 1–2: purified rTGH4-m. **C** The ratio of the area of migrating cells to the scratched area. **D** Fluorescence intensity diagram and gating of proliferating FITC^int^ cells. **E** Statistical analysis of the percentage of proliferating FITC^int^ cells. **F** Western blot analysis of intracellular effector proteins in fibroblasts treated with rTGH4 and rTGH4-m. **G** Relative quantitative statistical analysis of protein expression. The data are presented as the mean ± SD from three independent experiments. * P < 0.05, ** P < 0.01, *** P < 0.001, ns: no significant difference. (Two-way ANOVA followed by Dunnett’s multiple comparisons test and represented as * [**C**, **G**]; one-way ANOVA followed by Dunnett’s multiple comparisons test and represented as * [**E**]).

Considering that signalling pathway studies in worms are not feasible and that the active regions of BMP family protein sequences are highly conserved among different species, we used mouse fibroblasts in vitro to investigate whether rTGH4 and rTGH4-m have BMP activity. The recombinant proteins were successfully obtained, the molecular weights were consistent with the prediction, and no other obvious proteins were present (Figure 2A). Interestingly, the results of native PAGE revealed that the molecular weight of rTGH4-m identified by an anti-His tag antibody was 25–30 kDa, suggesting that soluble rTGH4-m is similar to the BMP family in the form of dimers (Figure 2B). Next, we examined cell migration and proliferation to determine the cell state. The results revealed that rTGH4 and rTGH4-m inhibited cell migration (rTGH4: *P* < 0.01; rTGH4-m: *P* < 0.01) and proliferation (rTGH4: *P* < 0.001; rTGH4-m: *P* < 0.001) (Figures 2C–E and Additional file 2). rTGH4-m can decrease the expression of collagen I (*P* < 0.001) and α-SMA (*P* < 0.001), which are two representative proteins related to the differentiation of fibroblasts [34]. Whether the regulatory effects of proteins on cells depend on the BMP signalling pathway was examined. Interestingly, rTGH4 cannot induce the phosphorylation of Smad1/5 (*P* > 0.05) but can inhibit the phosphorylation of p38-MAPK (*P* < 0.001), which may be related to the inhibition of cell proliferation and migration (Figures 2F, G). In contrast, rTGH4-m can induce the phosphorylation of p38-MAPK (*P* < 0.05) and Smad1/5 (*P* < 0.001) and inhibit the phosphorylation of Smad2/3 (*P* < 0.001), which directly demonstrates that rTGH4-m can activate the BMP signalling pathway in mice.

### TGH4 is the key candidate vaccine antigen related to the growth and development of newborn larvae (NBL)

**Figure 3:**
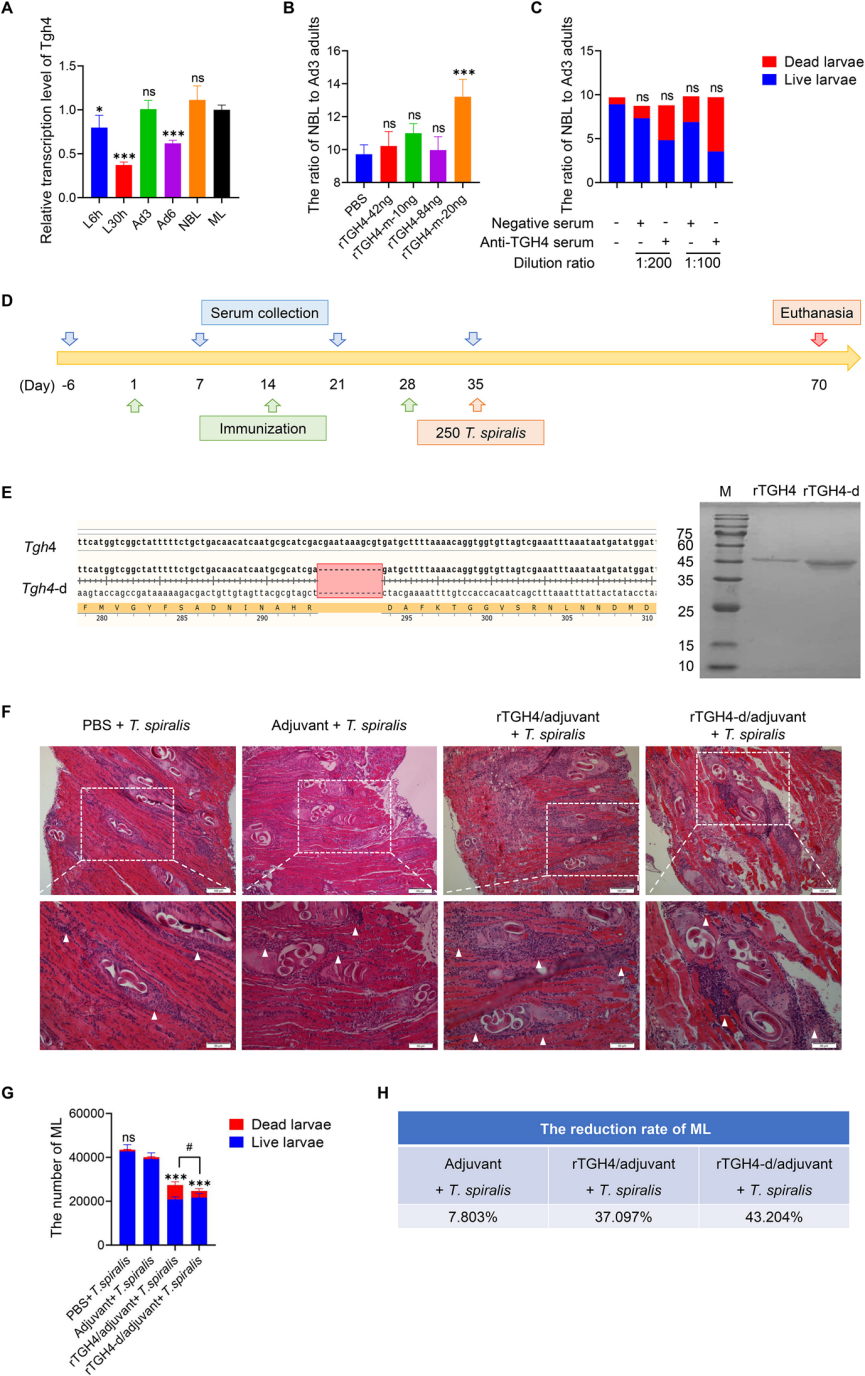
**TGH4 affects embryonic development and larval parasitism.** **A** Transcriptional levels of Tgh4 in different developmental stages of *T. spiralis*. **B** Effects of rTGH4 and rTGH4-m on the generation of NBL from Ad3 adults. **C** Effect of anti-TGH4 serum on the generation of NBL from Ad3 adults. **D** Schematic diagram of the immune protection experimental protocol. **E** Design of the mutant protein rTGH4-d without the furin cleavage site and SDS-page of rTGH4 and rTGH4-d. **F** H&E-stained sections of the mouse diaphragm. The lower image is a partial enlargement of the white area of the above image. The white arrow indicates the infiltration of inflammatory cells. **G** The amounts of total ML in the mice, including dead and living ML. **H** The average reduction rate of ML in each group. The data are presented as the mean ± SD from five independent experiments. *, #* P* < 0.05; **, ##* P* < 0.01; ***, ###* P* < 0.001; ns: no significant difference. (One-way ANOVA followed by Dunnett’s multiple comparisons test and represented as * [**A**, **B**, **C**, **G**]; unpaired t tests represented as # [**G**]).

To elucidate the relationship between TGH4 and *T. spiralis* development, the Tgh4 transcription levels of the worms at different time points of infection were first analysed. As expected, Tgh4 transcription levels were high at every growth and development stage, especially at the Ad3, NBL, and ML stages (Figure 3A). Next, the fertility of Ad3 adults was analysed by the ratio of NBL to Ad3 adults in vitro to understand the effect of BMP protein activity on the growth and development of embryos. We found that rTGH4-m had the ability to increase the ratio of NBL to adults, which increased with increasing protein dose (Figure 3B). These findings revealed that rTGH4-m can promote the development of embryos into larvae. Next, after three rounds of rTGH4 immunization, the serum was used to block the regulatory effects of natural TGH4 on the growth and development of larvae. However, anti-TGH4 serum had no effect on the ratio of NBL to Ad3 adults (*P* > 0.05), but it induced more dead larvae, which suggested that blocking natural TGH4 affected the survival of the larvae (Figure 3C).

We next investigated the biological effects of TGH4 loss on natural parasitic processes through active immunity (Figure 3D). Both worm and fibroblast experiments revealed that the released C-terminal TGH4-m was the key to exerting protein activity and that the furin cleavage site of TGH4 was the key to the release of active TGH4-m. rTGH4-m cannot be used as a vaccine antigen to induce specific antibodies (Additional file 3A). In addition, rTGH4-m may be released from rTGH4 to regulate the immune system and affect the active immune system during the immune program, and rTGH4-d without the furin cleavage site was therefore used as an inactive protein control (Figure 3E). Compared with those in the PBS and adjuvant control groups, many inflammatory cells infiltrated the cysts in the muscle tissue of the protein-immunized groups (Figure 3F). In addition, the parasite loads of ML were significantly lower in the protein-immunized groups than in the adjuvant control groups (rTGH4: *P* < 0.001; rTGH4-d: *P* < 0.001), especially in the rTGH4-d-immunized group (Figures 3G and H). Interestingly, the protein-immunized groups presented an increased number of dead larvae, suggesting that the activity of the larvae was impaired during the formation of cysts. The significant difference in total ML counts between the two protein-immunized groups (*P* < 0.05) was caused mainly by the reduced number of dead larvae in the rTGH4-d-immunized group.

### rTGH4-d induces changes in serum antibody IgG subclasses to affect the parasitism of NBL

**Figure 4:**
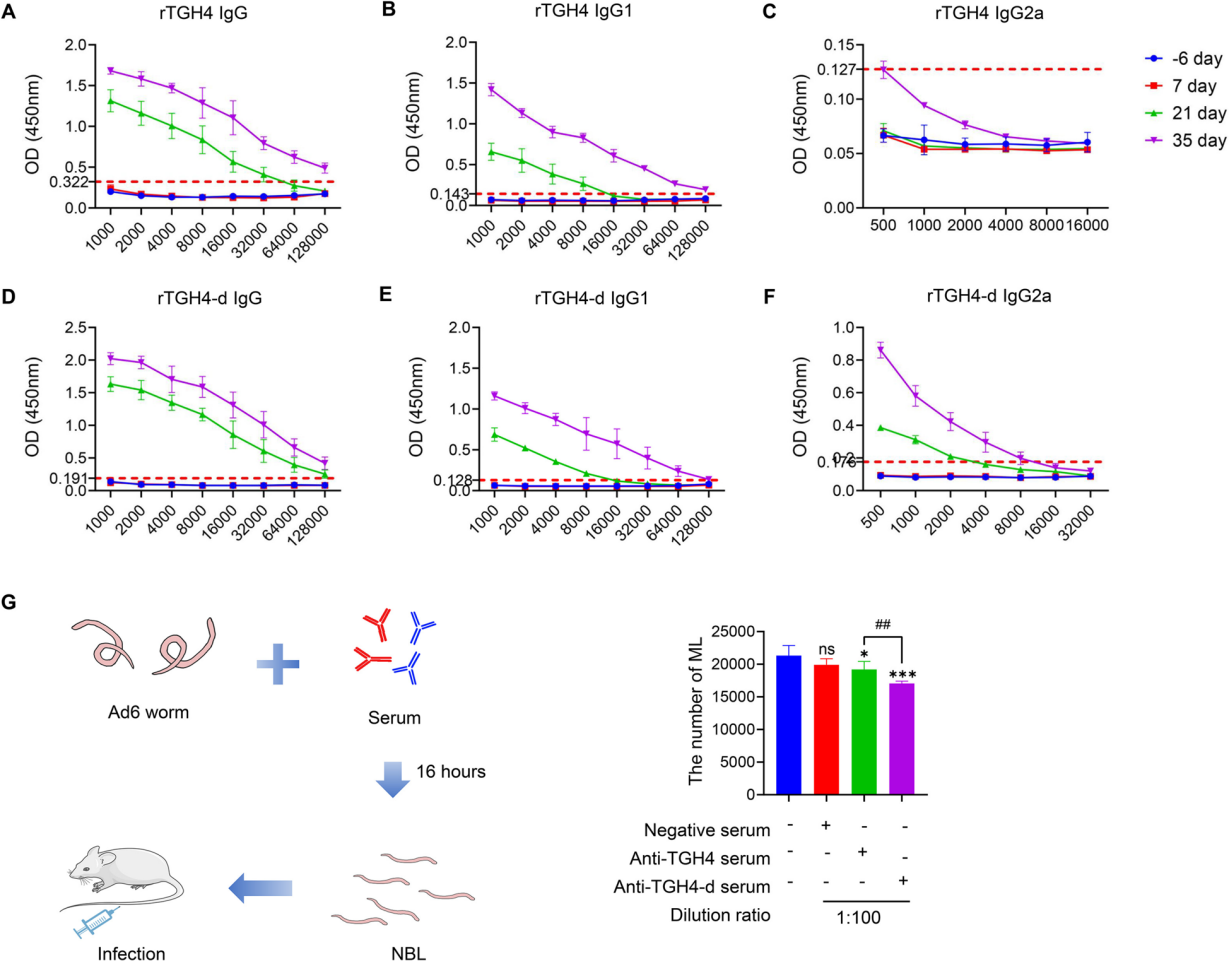
**rTGH4-d induces different specific antibody subclasses to affect the parasitism of NBL.** **A**–**C** Specific IgG, IgG1, and IgG2a antibody levels in the serum of rTGH4-immunized mice at different time points. **D**–**F** Specific IgG, IgG1, and IgG2a antibody levels in the serum of rTGH4-d-immunized mice at different time points. **G** Ad6 adults were incubated with negative serum/anti-TGH4 serum/anti-TGH4-d serum for 16 h to collect NBL, and the infective activity of NBL was detected by infecting the mice. The data are presented as the mean ± SD from five independent experiments. *, #* P* < 0.05; **, ##* P* < 0.01; ***, ###* P* < 0.001; ns: no significant difference. (One-way ANOVA followed by Dunnett’s multiple comparisons test and represented as * [**G**]; unpaired t tests represented as # [**G**]).

Having established a blood collection time during the immune program, we investigated the changes in the levels of different specific antibody subclasses in the serum. Protein and Freund’s adjuvant immunization did not increase the level of specific IgA in the serum (Additional file 3B). The antibody titres of IgG in the serum increased with the number of immunizations, and the serum titres were all higher than 128 000 after three immunizations (Figures 4A and D). Interestingly, rTGH4 induced significantly high levels of IgG1 and low levels of IgG2a, suggesting that it could mainly induce type II immune responses (Figures 4B and C). In contrast, rTGH4-d significantly induced the production of specific IgG2a, suggesting that it induced a mixture of type I and type II immune responses (Figures 4E and F). To gain a more in-depth understanding of the effects of changes in antibodies, NBL were incubated with serum enriched with protein three times, and reinfection experiments were performed (Figure 4G). Compared with the negative control serum, the protein-immunized serum significantly reduced the amount of ML (rTGH4: *P* < 0.05; rTGH4-d: *P* < 0.001), especially the rTGH4-d-immunized serum.

### rTGH4 and rTGH4-d induce different changes in the phenotypes of immune cells in the spleen

**Figure 5:**
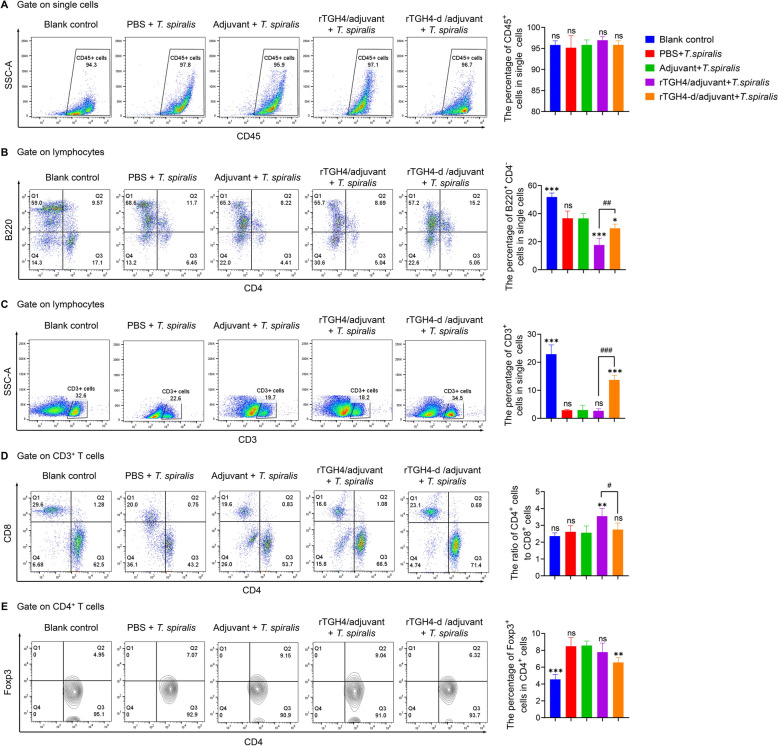
**rTGH4 and rTGH4-d have different abilities to regulate immune cells in the spleen.** Representative dot plots and statistical analysis of **A** CD45^+^ immune cells, **B** B220^+^CD4^−^ B cells, **C** CD3^+^ T cells, **D** CD4^+^ T and CD8^+^ T cells, and **E** CD4^+^ Foxp3^+^ Treg cells. The data are presented as the mean ± SD from five independent experiments. *, #* P* < 0.05; **, ##* P* < 0.01; ***, ###* P* < 0.001; ns: no significant difference. (One-way ANOVA followed by Dunnett’s multiple comparisons test and represented as * [**A**–**E**]; unpaired t tests represented as # [**B**–**D**]).

To understand immune changes during infection, we detected changes in immune cells in the spleen on day 70. The proportion of total immune cells was not significantly different among the groups (*P* > 0.05), but the cellular phenotype changed (Figure 5A and Additional file 4). The number of B cells in the spleen was significantly lower in the protein-immunized groups than in the adjuvant control groups (rTGH4: *P* < 0.001; rTGH4-d: *P* < 0.05) (Figure 5B). T cells are the critical effector cells of adaptive immunity in active immune processes, including the direct killing effect of CD8^+^ cells and the auxiliary enhancement of the immune response of CD4^+^ cells [35, 36]. Interestingly, rTGH4 and rTGH4-d affected the proportion and differentiation of T cells in two different ways. In the absence of changes in the proportion of CD3^+^ T cells (*P* > 0.05), compared with the adjuvant control, rTGH4 increased the ratio of CD4^+^ cells to CD8^+^ cells (*P* < 0.01) to enhance the immune response and resistance against *T. spiralis* (Figures 5C–E). However, compared with the adjuvant control group, the rTGH4-d group presented a high proportion of CD3^+^ T cells (*P* < 0.001) and a low proportion of Treg cells (*P* < 0.01), but the ratio of CD4^+^ T cells to CD8^+^ T cells remained unchanged (*P* > 0.05).

### rTGH4 and rTGH4-d affect macrophages in the peritoneal cavity to enhance the immune response

**Figure 6:**
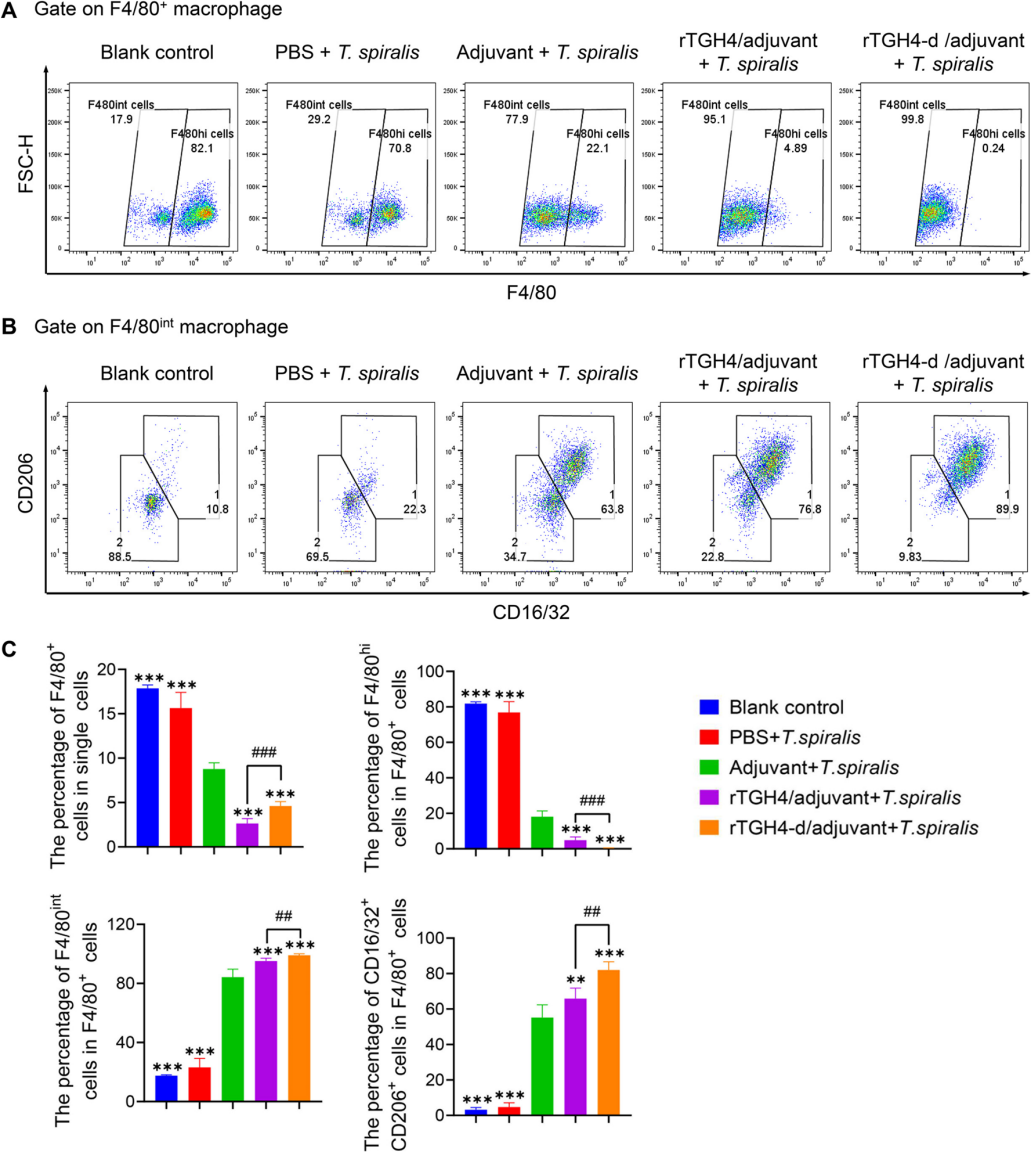
**rTGH4-d recruits more monocyte-derived macrophages into the peritoneal cavity than does rTGH4.** Representative dot plots of **A** F4/80^hi^ macrophages and F4/80^int^ macrophages and **B** F4/80^int^ CD16/32^+^ CD206^+^ macrophages. **C** The percentage of total F4/80^+^ macrophages in single cells, the percentage of F4/80^hi^ macrophages in F4/80^+^ macrophages, the percentage of F4/80^int^ macrophages in F4/80^+^ macrophages, and the percentage of F4/80^int^ CD16/32^+^ CD206^+^ macrophages in F4/80^+^ macrophages. The data are presented as the mean ± SD from five independent experiments. *, #* P* < 0.05; **, ##* P* < 0.01; ***, ###* P* < 0.001; ns: no significant difference. (One-way ANOVA followed by Dunnett’s multiple comparisons test and represented as *; unpaired t tests represented as #).

In normal physiological homeostasis, macrophages in the peritoneal cavity are composed mainly of F4/80^hi^ large macrophages and F4/80^int^ small macrophages derived from monocytes [37, 38] (Additional file 4). Compared with that in the adjuvant control group, the number of large macrophages significantly decreased in the protein-immunized groups (rTGH4: *P* < 0.001; rTGH4-d: *P* < 0.001), and the number of small macrophages significantly increased (rTGH4: *P* < 0.001; rTGH4-d: *P* < 0.001), suggesting that the immune state in the peritoneal cavity was strongly related to the inflammatory response (Figures 6A–C). Compared with rTGH4, rTGH4-d has a greater ability to induce the differentiation of small macrophages and the expression of CD16/32 and CD206 (*P* < 0.01), which are key membrane proteins that take up and process antigens and initiate an adaptive immune response [39, 40].

### TGH4-d induces high levels of proinflammatory cytokines

**Figure 7:**
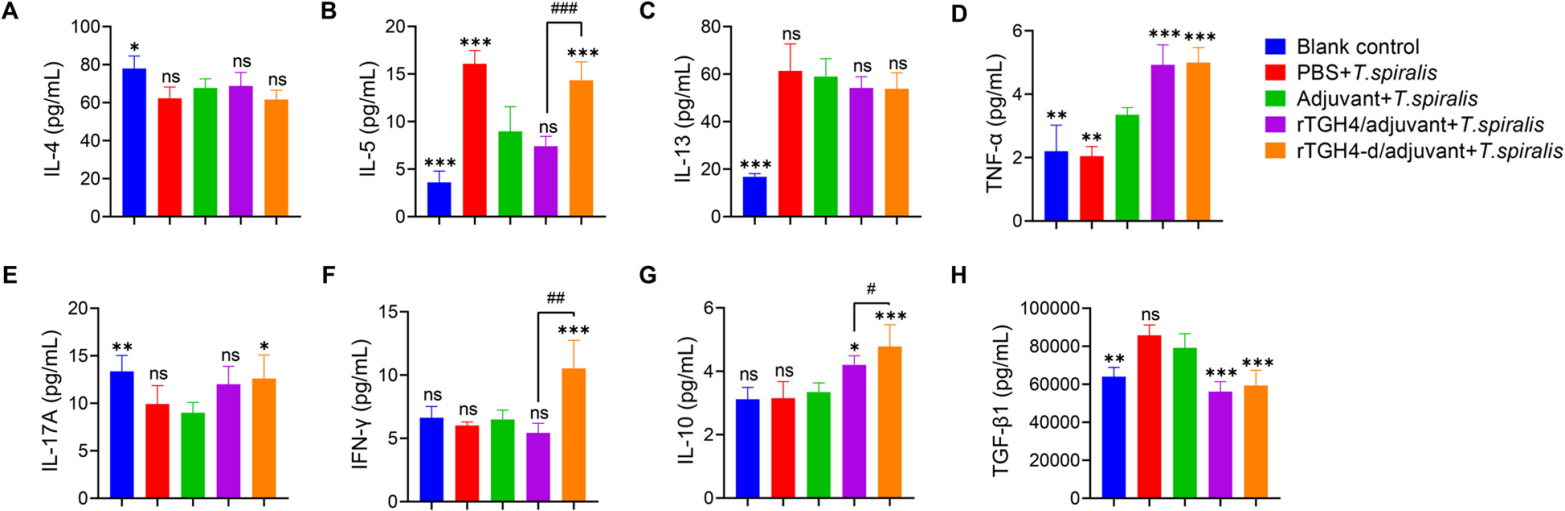
**Detection of the pro-/anti-inflammatory cytokine levels in the serum.** The IL-4, IL-5, IL-13, TNF-α, IL-17A, IFN-γ, IL-10, and TGF-β1 levels in the serum were detected on day 70. The data are presented as the mean ± SD from five independent experiments. *, #* P* < 0.05; **, ##* P* < 0.01; ***, ###* P* < 0.001; ns: no significant difference. (One-way ANOVA followed by Dunnett’s multiple comparisons test and represented as * [**A**–**H**]; unpaired t tests and unpaired t tests are represented as # [**B**, **F**, **G**]).

To complement the above findings, cytokine levels in the serum, which can reflect the systemic immune status, were measured. Among the three typical type 2 immune-related cytokines, the protein-immunized groups presented no significant differences in interleukin (IL)−4 levels (rTGH4: *P* > 0.05; rTGH4-d: *P* > 0.05) or IL-13 levels (rTGH4: *P* > 0.05; rTGH4-d: *P* > 0.05) compared with the adjuvant control group, but rTGH4-d induced higher IL-5 levels than did rTGH4 (*P* < 0.001) (Figures 7A–C). The IL-17A (rTGH4: *P* > 0.05; rTGH4-d: *P* < 0.05) and TNF-α (rTGH4: *P* < 0.001; rTGH4-d: *P* < 0.001) levels in the protein-immunized groups were greater than those in the adjuvant group, suggesting that active immunity with proteins can induce a strong inflammatory response (Figures 7D and E). In accordance with the antibody results, the IFN-γ level was significantly increased in the rTGH4-d-immunized group (*P* < 0.001) but not in the rTGH4-immunized group (*P* > 0.05), which confirmed that the type I immune response was crucial for the production of IgG2a (Figure 7F). Interestingly, the two typical anti-inflammatory cytokines showed opposite trends. The protein-immunized groups presented reduced TGF-β1 levels (rTGH4: *P* < 0.001; rTGH4-d: *P* < 0.001) and increased IL-10 levels (rTGH4: *P* < 0.05; rTGH4-d: *P* < 0.001), suggesting that these two cytokines may be produced by cells with different functions (Figures 7G and H).

## Discussion

We identified a BMP homologue, TGH4, from *T. spiralis* and demonstrated that active TGH4-m released from TGH4 was necessary to exert BMP activity. The conservative regulatory activity of rTGH4-m can not only maintain the resting state of mouse fibroblasts but also improve the reproductive ability of Ad3 adults. The active immunity of rTGH4 and rTGH4-d without an activated potential suggests that BMP activity has a negative effect on establishing effective immune protection against NBL parasitism. We confirmed that the protein activity of immunogens affects immune initiation and development during active immunity.

Several studies have confirmed that decreased expression of BMP6 and BMP7 is associated with fibrosis and cancer cell proliferation [41, 42]. BMP6 signalling has the ability to limit the development of liver fibrosis in mice and humans and reverse the fibrosis of HK-2 cells induced by TGF-β [43, 44]. Like BMP6, rTGH4-m has the ability to induce mouse fibroblasts to maintain a resting state with low proliferation and weak differentiation by activating the BMP signalling pathway. These results confirmed that the interaction site of the active region TGH4-m with the mouse BMP receptor was conserved. Therefore, cross-reactivity in the host induced by TGH4-m needs to be avoided in subsequent vaccine studies, which provides direction for protein mutation schemes. Interestingly, although rTGH4 can induce a similar resting state in cells, it does not depend on BMP signalling. The inhibition of the p38-MAPK signalling pathway may explain why TGH4 exerts similar effects, but the exact mechanism is still not understood.

The correlation between the protein and the growth and development of worms is the key to screening candidate vaccine antigens. Ad3 female adults of *T. spiralis* are key for the study of embryo development and artificial intervention in vitro. *T. spiralis* can reach sexual maturity to complete mating and the formation of fertilized eggs within 30 h of infection, which determines the maximum number of NBL. Hence, the BMP activity of rTGH4-m increased the ratio of NBL to Ad3 adults primarily by affecting embryonic development. When natural TGH4 was blocked with an anti-TGH4 antibody, the mortality of larvae significantly increased, which suggests that affecting embryo development may be a part of vaccine protection. The Freund adjuvant widely used in scientific research can assist proteins in inducing a strong systemic immune response that is necessary to study larval parasitism in different tissues [45]. Given that TGH4-m may regulate the host BMP pathway, the furin cleavage site was removed to prevent its release and regulatory effects. The rTGH4-d-immunized mice not only had a low parasite load but also had fewer dead larvae with cysts. Notably, the heterologous substances released from the disintegration of dead ML can cause strong inflammation [46], so stripping protein activity protects the mice from damage caused by excessive muscle inflammation.

How does the protein activity of TGH4 regulate active immunity to affect *T. spiralis* parasitism? Specific antibodies are key effector molecules that maintain long-term protection, and their subclasses can reflect the state of the host immune system [47, 48]. Our study revealed that stripping protein activity can change the specific IgG2a level, which is consistent with an increase in the IFN-γ level in the serum. IgG2a can kill invading pathogens by activating the complement system, which contributes to reducing the number of schistosomula recovered from the lungs in a *Schistosoma mansoni* infection model [49, 50]. Our findings again provide evidence that specific IgG2a is key for reducing larval parasitism. In addition, the resistance of mature NBL to anti-TGH4 serum indirectly proves that blocking BMP signalling is important for embryonic development.

Cellular immunity affects the development direction of humoral immunity. To better understand the effects of protein activity on the immune system, we analysed the changes in various immune cells in the spleen. Our study revealed that rTGH4 can increase the proportion of CD4^+^ T cells to enhance the immune response. However, stripping protein activity can increase the proportion of total T cells, which is associated with increased levels of IL-5, IFN-γ, and IL-10 in the serum. In addition, rTGH4-d can recruit more monocytes into the peritoneal cavity and induce them to differentiate into macrophages, which have a stronger ability to activate the adaptive immune response. In general, stripping protein activity can strongly induce the activation of innate and adaptive immunity, thus enhancing the humoral immune response and host resistance to *T. spiralis*.

Our work first identified the BMP homologue TGH4 from *T. spiralis* and demonstrated that the C-terminal active region of TGH4-m released from TGH4 was the key to exerting BMP activity. Similar to the function of BMP family proteins, the BMP activity of rTGH4-m is the key to promoting embryonic development. Two ways in which vaccines exert their protection are by affecting the embryonic development and migration of NBL. In addition, TGH4-d without BMP activity builds strong immune resistance by regulating the levels of macrophages, T cells, B cells, and specific antibodies. Modifying proteins to eliminate BMP activity, which plays a negative role in protection efficiency, provides a paradigm reference for vaccine modification of BMP homologues from other parasites.

## Supplementary Information

**Additional file 1. Amino acid sequence of TGH4.** The purple amino acids are signalling peptides. The blue amino acids are the cleavage sites of Furin. The brown labelled amino acid is TGH4-m. The red amino acid is the conserved cysteine of TGH4-m.

**Additional file 2. Images of the wound healing assays at different time points.**

**Additional file 3. The detection of specific antibodies in the serum of protein-immunized mice. **Western blot analysis of rTGH4-m incubated with negative control serum or anti-TGH4-m serum. The specific IgA level in the serumwas detected by ELISA.

**Additional file 4. Representative gating strategy for lymphocytes in the spleen and macrophages in the peritoneal cavity. **Representative flow cytometry plots of CD45^+^ immune cells, B220^+^ CD4^−^ B cells, CD4^+^ T cells and Foxp3^+^ Treg cells in the spleen are shown. Representative flow cytometry plots of CD3^+^ T cells, CD4^+^ T cells, and CD8^+^ T cells are shown.Total F4/80^+^ cells, F4/80^hi^ cells, F4/80^int^ cells, and CD16/32^+^ CD206^+^ macrophages in the peritoneal cavity were identified.

## Data Availability

The data of this study are available from the corresponding author upon reasonable request.

## Abbreviations

*T.** spiralis*: *Trichinella spiralis*
ML: muscular larvae
NBL: newborn larvae
Treg cells: T regulatory cells
BMP: bone morphogenetic protein
IL: interleukin
CFSE: carboxyfluorescein succinimidyl ester
