# Supplementary figures and images for: The unique activity of the bone morphogenetic protein TGH4 affects the embryonic development of Trichinella spiralis and the establishment of vaccine protection
Source: Vet Res. 2025 Feb 7;56:31. doi: 10.1186/s13567-025-01473-4 (PMC11803935; doi:10.1186/s13567-025-01473-4)

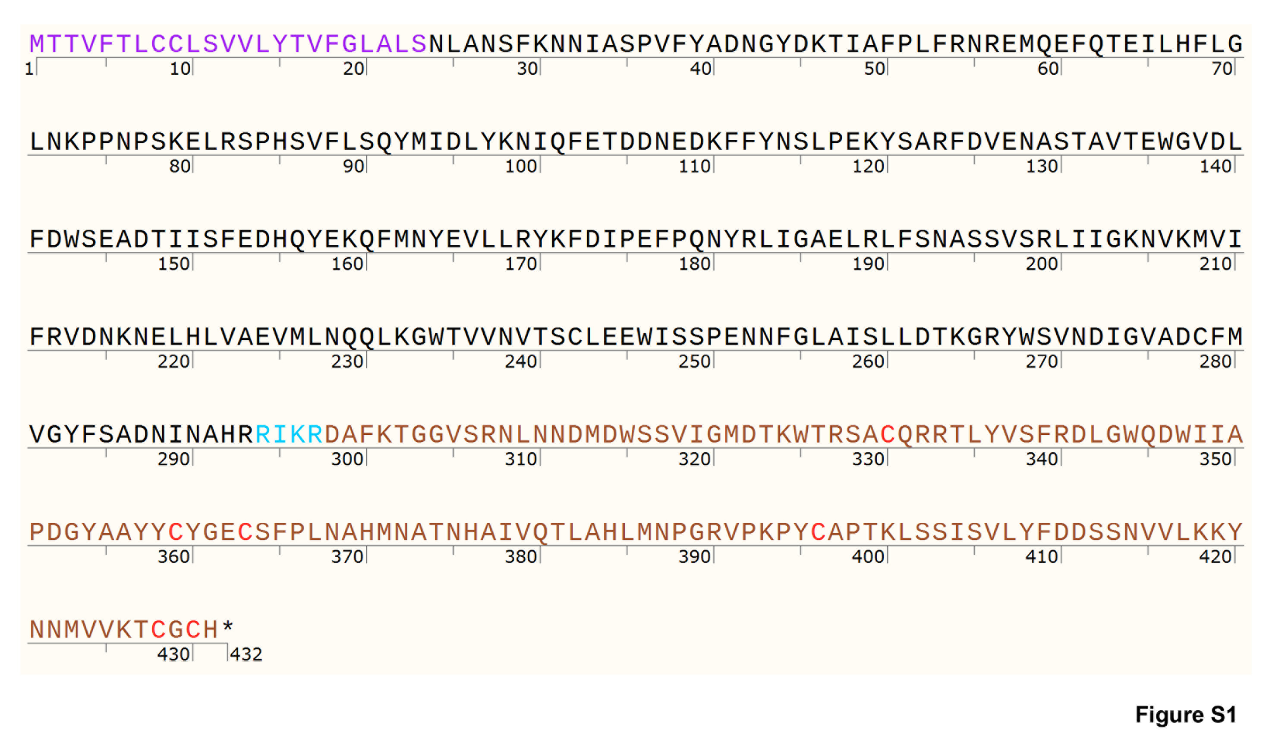

Supplement: Supplementary file 1 — Additional file 1. Amino acid sequence of TGH4. The purple amino acids are signalling peptides. The blue amino acids are the cleavage sites of Furin. The brown labelled amino acid is TGH4-m. The red amino acid is the conserved cysteine of TGH4-m. [file 13567_2025_1473_MOESM1_ESM.docx]

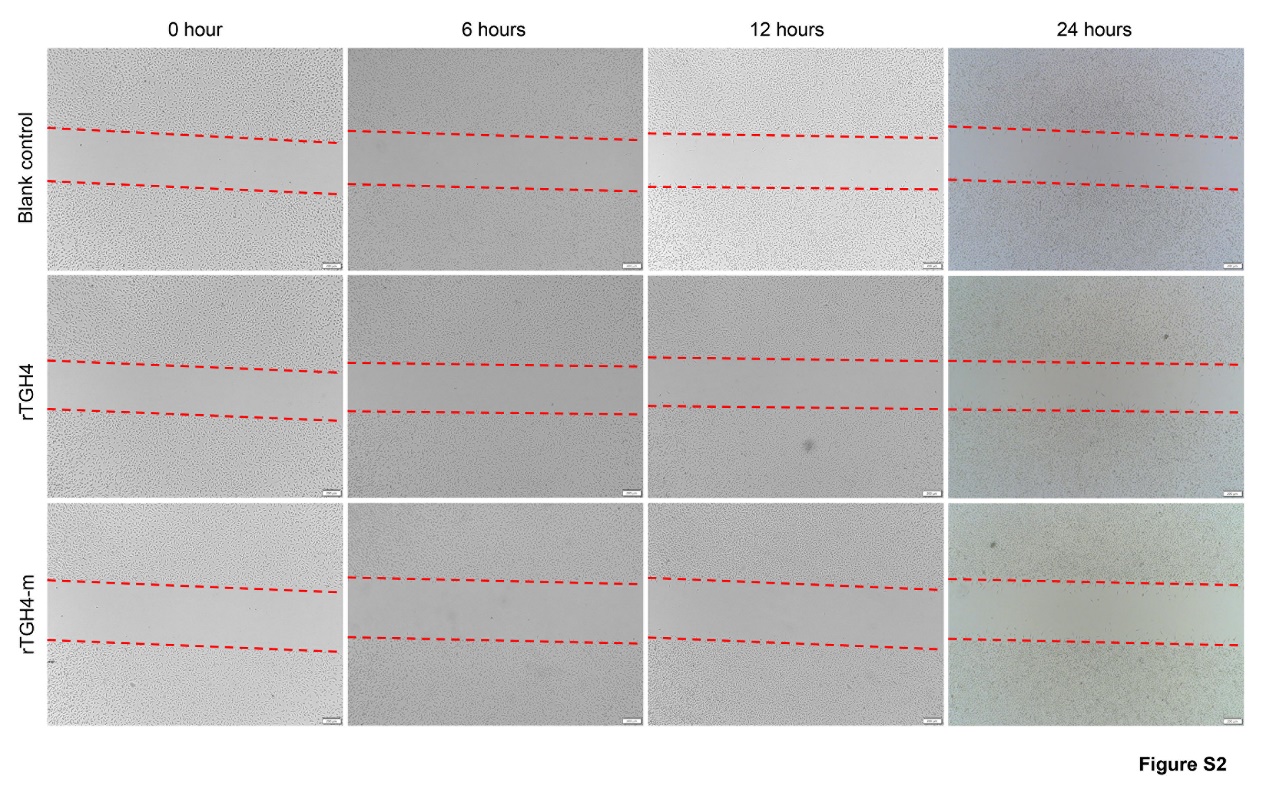

Supplement: Supplementary file 2 — Additional file 2. Images of the wound healing assays at different time points. [file 13567_2025_1473_MOESM2_ESM.docx]

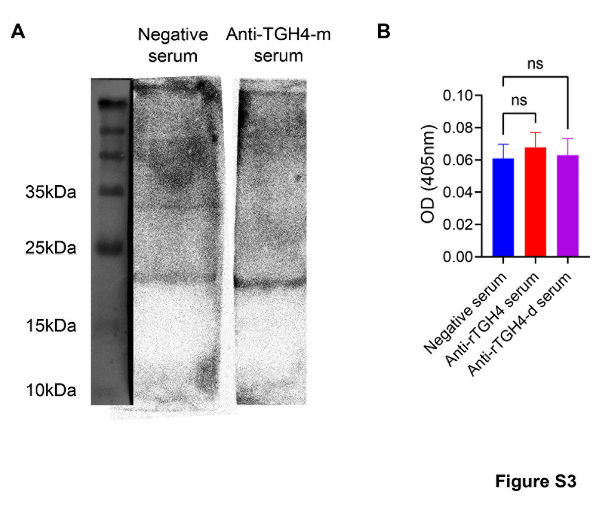

Supplement: Supplementary file 3 — Additional file 3. The detection of specific antibodies in the serum of protein-immunized mice. Western blot analysis of rTGH4-m incubated with negative control serum or anti-TGH4-m serum. The specific IgA level in the serumwas detected by ELISA. [file 13567_2025_1473_MOESM3_ESM.docx]

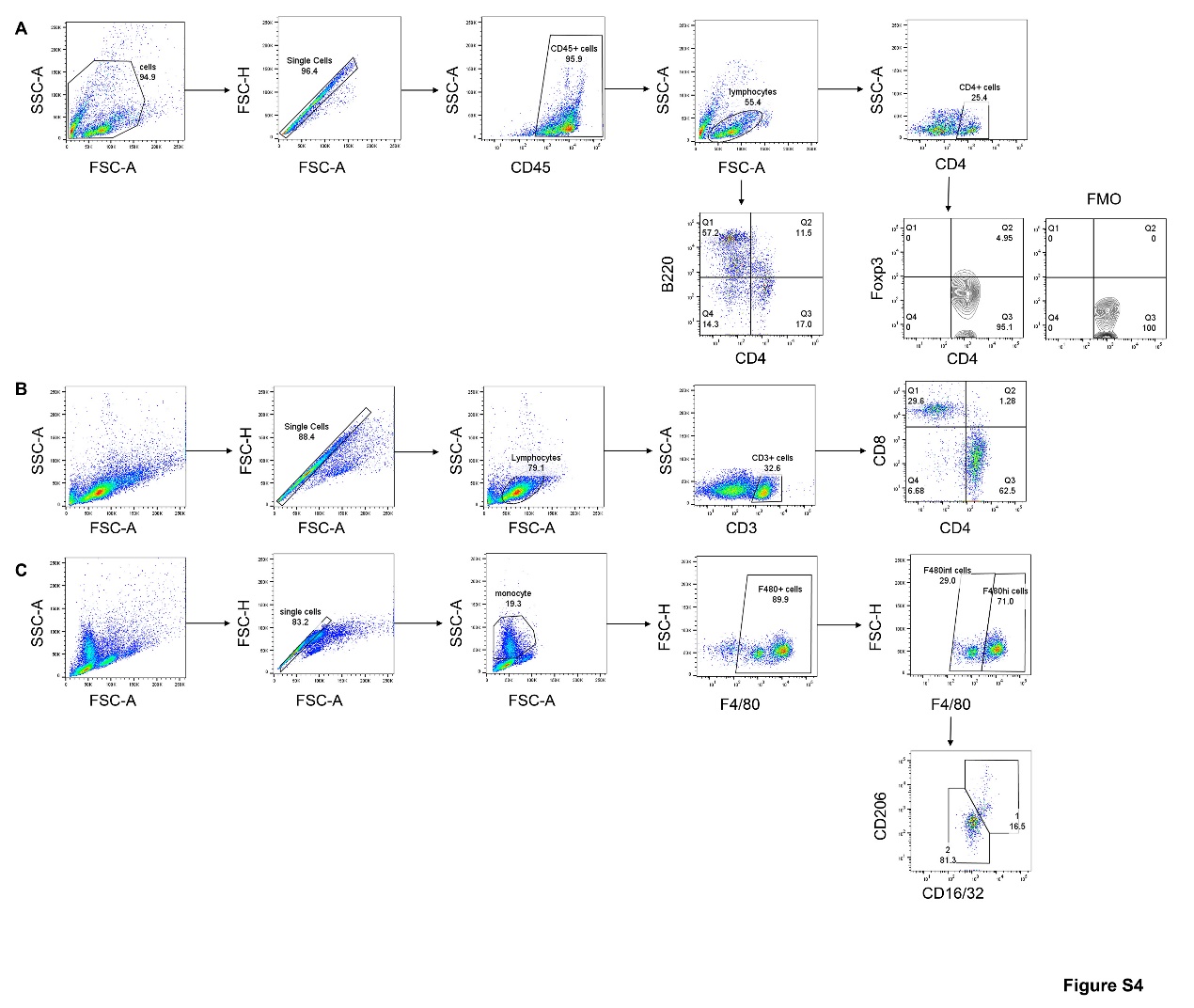

Supplement: Supplementary file 4 — Additional file 4. Representative gating strategy for lymphocytes in the spleen and macrophages in the peritoneal cavity. Representative flow cytometry plots of CD45+ immune cells, B220+ CD4− B cells, CD4+ T cells and Foxp3+ Treg cells in the spleen are shown. Representative flow cytometry plots of CD3+ T cells, CD4+ T cells, and CD8+ T cells are shown.Total F4/80+ cells, F4/80hi cells, F4/80int cells, and CD16/32+ CD206+ macrophages in the peritoneal cavity were identified. [file 13567_2025_1473_MOESM4_ESM.docx]
